# Editorial: Circadian Plasticity—A Collaboration Between Neuronal and Glial Oscillators

**DOI:** 10.3389/fphys.2019.00951

**Published:** 2019-07-30

**Authors:** Jolanta Górska-Andrzejak, Elżbieta Pyza

**Affiliations:** Department of Cell Biology and Imaging, Institute of Zoology and Biomedical Research, Jagiellonian University, Kraków, Poland

**Keywords:** circadian rhythms, biological clock, clock genes, oscillators, glial cells, astrocytes, plasticity, neuron-glia interactions

The amounts of glia in the brains of *Drosophila* (25%), rodents (65%), and humans (90%) suggest that increasing complexity of the brain is accompanied by growing numbers of glial cells (Losada-Perez, 2018). In view of this observation it comes as no surprise that glial cells that were once perceived merely as the supportive constituent for neurons are currently regarded as neuronal partners and important contributors to neuronal circuits formation and functioning.

This Research Topic focuses on the team work of neurons and glial cells in the oscillatory systems. It mostly focuses on glial contribution to the circadian rhythms that adapt organisms to the solar cycle, but it also explores the role of glia in the brain oscillator that drives discharge of the electric organ of the weakly electric fish, *Apteronotus leptorhynchus* (Zupanc). The latter has recently emerged as an excellent model of the oscillatory network (Sîrbulescu et al., 2014; Zupanc et al., 2014). This collection of articles gives new information on the still elusive role played by glial cells in such networks and reviews our current understanding of glial functioning in oscillatory systems.

A review paper by Chi-Castañeda and Ortega recapitulates the current information on the role of glial cells in the circadian regulation of synaptic plasticity, exploiting its genetic, molecular, and physiological aspects. The authors provide an overview of literature that not only links circadian pacemakers with glial function but also gives the clinical implication of circadian clock and glial dysfunctions in diverse brain pathologies.

Another in-depth review, given by Lindberg et al. links the purinergic signaling to both circadian clock function and alcohol use disorder (AUD), as the normal diurnal oscillation of the key neurotransmitters like glutamate, GABA, dopamine and ATP adenosine, which underlie the circadian homeostasis, can be disrupted by excessive alcohol consumption. Considering the regulation of purinergic signaling and circadian oscillations by both neurons and astrocytes, as well as their interactions, they review the diverse mechanisms by which purinergic malfunction may contribute to circadian disruption or alcohol abuse.

Studies by Duhart et al. show effects of human glioma cells (the primary brain tumor with the highest incidence and mortality) in the hypothalamic region on the circadian behavioral output in mice. Their report might be of relevance for glioma diagnosis as it provides the foundation work for future research aimed to understand the pathological consequences of astrocytic dysfunction in the circadian time-keeping, which have recently come to light as a major player in SCN (Brancaccio et al., 2017; Tso et al., 2017).

The research by Guissoni Campos et al., on the other hand, contributes to current knowledge of rhythmic characteristics of the cerebellar cortex (the major center of motor activity), including the neuron-glia interactions that may impact the processing in the cerebellum. The authors show possible relation between neurons expressing the clock protein PERIOD (which is a hallmark of a functioning molecular oscillator; PER) and Bergmann glia, which are demonstrated to express melatonin receptors.

Functioning of both neuronal and glial oscillators is the foundation of the circadian plasticity of the visual system of *Drosophila melanogaster* (Górska-Andrzejak, 2013). The studies on *Drosophila* included in this Research Topic reveal the heterogeneity of glial oscillators in the *Drosophila* optic lobe. This line of research has opened new perspectives in studying the glial circadian clock with regard to different subtypes of glial cells (Górska-Andrzejak et al.; Krzeptowski et al.). Górska-Andrzejak et al. bring attention to the glial cells located in the output of the circadian pacemaker in the neuropil of optic medulla and infer the possibility of interactions between Pigment Dispersing Factor (PDF) releasing clock neurons and the medulla glia expressing PDF receptors. Further characterization of the distal medulla glia by Krzeptowski et al. reveals two populations of cells that differ with respect to expression level of PER and the glial marker REPO. Interestingly, the authors have observed that the elevated levels of PER are characteristic for the ensheathing glia, but not the astrocyte-like ones (no staining of PER in the astrocyte-like glia was also reported by Long and Giebultowicz), even though the latter are well-known to influence the circadian rhythms of *Drosophila* locomotor activity (Suh and Jackson, 2007; Ng et al., 2011). It has to be taken into account, therefore, that the glial cells with low level of PER may also play a role in *Drosophila* circadian network (Krzeptowski et al.). Long and Giebultowicz additionally demonstrate that the rhythmic expression of PER dampens with advanced age in most of the glial subtypes. Thus, their study bring into focus the glia-related circadian changes that may significantly contribute to the loss of homeostasis in the aging brain.

Another study on *Drosophila* (Herrero et al.) emphasizes the important role of neuron-glial interactions in the structural plasticity of the circadian network. The article by Ceriani group (Herrero et al.) reveals the involvement of glial cells in the structural remodeling of neuronal pacemakers (Fernández et al., 2008), which changes the degree of the circadian network connectivity during the day. In this research, they have found that the molecular clocks in both clock neurons and glia are required for sustaining the circadian structural plasticity of the clock neuron projections. Yet another model of the pacemaker nucleus in *A. leptorhynchus* also reveals glial involvement in the plasticity of neuronal oscillators (Zupanc). It explains how the dynamic morphological changes of dense astrocytic meshwork might modulate the output activity of the neuronal oscillators to produce dimorphic behavior of females and males.

Eventually, the last paper on *Drosophila* (Cusumano et al.) corroborates the hypothesis that the circadian clock may also adopt post-transcriptional mechanisms regulating transposable elements (TEs) in order to ensure proper rhythmicity. Its authors argue that BELLE, a conserved DEAD-box RNA helicase that acts as an important piRNA-mediated regulator of TEs, is a putative clock component present in both the clock neurons and the glial cells of *Drosophila* brain (Cusumano et al.) and influences circadian rhythmicity of *Drosophila* locomotor activity.

## Author Contributions

All authors listed have made a substantial, direct and intellectual contribution to the work, and approved it for publication.

### Conflict of Interest Statement

The authors declare that the research was conducted in the absence of any commercial or financial relationships that could be construed as a potential conflict of interest.
